# West Nile Virus Detection in American Crows

**DOI:** 10.3201/eid0910.030306

**Published:** 2003-10

**Authors:** Sarah A. Yaremych, Richard E. Warner, Marshall T. Van de Wyngaerde, Adam M. Ringia, Richard Lampman, Robert J. Novak

**Affiliations:** * University of Illinois, Urbana, Illinois, USA; † Illinois Natural History Survey, Champaign, Illinois, USA

## Abstract

A dipstick immunochromatographic assay used for West Nile virus (WNV) detection in mosquitoes was investigated for application to testing of fecal, saliva, and tissue samples from dead American Crows (*Corvus brachyrhynchos*). Results suggest that VecTest may be an efficient method for WNV detection in field-collected, dead American Crows, although confirmation of results and further investigation are warranted.

The American Crow (*Corvus brachyrhynchos)* has been designated as a West Nile virus (WNV) surveillance species (*1*), and dead American Crows have been used to monitor WNV activity across the nation. The American Crow is a useful species for monitoring disease activity because it is highly visible and recognizable; furthermore, all American Crows experimentally inoculated with WNV have died within 7 days of inoculation after attaining viremias of sufficient titer to infect mosquitoes (*2*). Avian deaths early in the transmission season are a warning for increased risk for human WNV cases (*3*); by monitoring WNV infection in dead American Crows, we can detect areas of epidemiologic public health concern.

Standard methods of identifying WNV in dead crows include two direct tests of tissues by immunohistochemistry (IHC) and reverse transcriptase-polymerase chain reaction (RT-PCR TaqMan (Applied Biosystems, Foster City, CA). Komar et al. (*4*) determined that postmortem cloacal and oral swabs could replace brain tissue as a specimen for WNV detection in crows and jays. Addressing this, and the need for a simple, quick, and cost-effective method for viral surveillance in dead crows, we conducted a study to determine whether the VecTest WNV/Saint Louis encephalitis virus (SLEV) Antigen Panel Assay (Medical Analysis Systems Inc., Camarillo, CA) could be used for WNV testing of fecal, saliva, and tissue samples from American Crows.

The VecTest was designed as a rapid wicking assay to identify the presence or absence of viral antigen specific to WNV or SLEV in infected mosquitoes. This test employs monoclonal antibodies against WNV and SLEV in a one-step procedure with a wicking test strip. All components necessary to carry out the test are provided in a kit, including vials, buffer solution, and dipsticks. The tests can be performed in the field, highly trained personnel and specialized equipment are not necessary, and results can be obtained quickly (<20 minutes). Each dipstick contains an internal positive control to indicate that the test has performed properly. After mosquitoes are ground in a buffer solution, a dipstick is inserted; WNV and SLEV antigen present in the mosquito slurry will bind to the specific antibody-colloidal gold conjugate. Antigen presence is indicated by a red line that develops in the test zone of the dipstick, specific to WNV or SLEV; pictures of dipsticks can be obtained from Ryan et al. (*5*). Ryan evaluated the product for detection of WNV in mosquitoes in a laboratory and suggested that sensitivity of VecTest is comparable to that of an antigen-capture enzyme-linked immunosorbent assay but less sensitive than Vero cell plaque assays or RT-PCR. Our study is a preliminary evaluation of the VecTest assay to determine whether it can be used for accurate WNV testing of field-collected dead American Crows.

## The Study

In February–October 2002, as part of a broader study, we captured, banded, and marked 156 American Crows (*C. brachyrhynchos*) in Champaign and Urbana, Illinois (Yaremych SA. West Nile virus and American Crows in east-central Illinois. University of Illinois; 2003). Radio-transmitters were attached to individuals of a subsample of captured crows. Many of the study crows died in the summer; all recovered crows were tested for WNV. Dead crows were retrieved by tracking the radio signal to the carcasses, by chance encounter, or by notification from the public. Dead study crows with transmitters were typically recovered <36 hours after death, as this period was the maximum amount of time elapsed between the last live observation of a crow during radio tracking and recovery of the carcass upon death. We estimated that other marked study crows without transmitters were retrieved up to 72 hours after death. The abundance of dead crows allowed for a simple comparative study involving the use of VecTest, RT-PCR, and IHC tests to detect WNV in crow fecal, saliva, and tissue samples.

For each dead crow, we diluted fecal scrapings from the cloaca and salivary scrapings from the mouth in VecTest buffer solution in the vials provided in the kit. A metal spatula was used to obtain samples, and instruments were sanitized with a wash of soap and water and 70% ethanol before and after use. In some cases, maggots were in such abundance in the mouth and cloaca that they could not be removed from the sample; they were added to the vials with the feces and saliva. For carcasses with no moist fecal or saliva sample, small metal scissors were used to clip any available dried internal tissues around the mouth and cloaca. These tissues were placed in the vials. Most vials contained a mixture of fecal, saliva, and tissue samples. Samples were shaken by hand for approximately 60 seconds for homogenization. From this point, we followed manufacturer’s directions, having replaced mosquitoes with fecal, saliva, and tissue samples. Indicator strips were inserted into the solution and interpreted in the field after 15 minutes.

Samples were stored at –80°C after we performed the test. To confirm the results, we used RT-PCR TaqMan to detect the presence or absence of WNV-RNA in these samples by a method similar to that used by Lanciotti et al. (*6*). A WNV strain (NY99) was used as a positive control. Alternatively, VecTest results were confirmed by IHC testing of the brain, heart, kidney, and spleen of the crow carcass from which the samples were derived. The IHC testing was conducted by the University of Illinois Veterinary Diagnostic Laboratory, according to the method outlined by Heinz-Taheny et al (*7*).

## Results

We used VecTest to test all 20 crow samples; all indicator strips developed control lines, which indicated that the test had performed according to instructions. Nineteen samples were positive for WNV with a faint-to-bold WNV line; one sample was negative for WNV. IHC testing was performed on the crow carcasses from which five of these positive samples were derived, and IHC labeling for WNV antigen was present in all five of these samples, indicating 100% confirmation of the VecTest results. The remaining 15 vials containing the VecTest crow sample, composed of 14 positives and 1 negative, were assayed by RT-PCR TaqMan. Results from TaqMan showed 11 positives and 4 negatives (Table). In total, 17 (85%) of 20 of the VecTest results were confirmed with either IHC or RT-PCR TaqMan, and 3 (15%) of 20 involved conflicting results between the two testing methods for the samples. No significant difference existed between the positive and negative rates of VecTest and RT-PCR in a chi-square analysis of a 2x2 contingency table (chi square = 2.16, df=1, p=0.14). Using RT-PCR as the standard criterion, we found that VecTest results included three false positives, yielding a false-positive rate of 75%.

**Table:** Results of West Nile virus testing of American Crow fecal, saliva, and tissue samples

Sample no.	VecTest^a^	RT-PCR TaqMan^b^
1	+	+
2	+	+
3	+	+
4	+	+
5	+	+
6	+	+
7	+	+
8	+	+
9	+	-
10	+	-
11	-	-
12	+	+
13	+	+
14	+	+
15	+	-

^a^VecTest West Nile virus/Saint Louis encephalitis virus Antigen Panel Assay (Medical Analysis Systems Inc., Camarillo, CA). ^b^Reverse transcriptase polymerase chain reaction; Taqman (Applied Biosystems, Foster City, CA).

## Conclusions

On the basis of their trials with mosquitoes, Ryan et al. (*5*) suggested that VecTest should not produce false-positive results. Although the rates of positives and negatives did not differ between VecTest and RT-PCR in this study, this result may be an artifact of small sample size. The false-positive VecTest results on American Crow fecal, saliva, and tissue samples suggest a low specificity; therefore, we recommend that VecTest be considered experimental in its application to dead American Crows until more extensive investigations are conducted. All positive VecTest results should be verified with another test. VecTest may be useful in early season screening, when rates of positives are typically low, or in nonpeak areas. We conducted this study in mid- to late summer in east-central Illinois, during a time when death rates of free-ranging American Crows were high.

RT-PCR detects genomic sequences of WNV, whereas VecTest detects the viral capsid. Because of the abundance of environmental RNAase and the possibility of the WNV capsid’s persisting longer than the RNA, the three false positives detected by VecTest may indeed contain WNV capsid. Alternately, the conjugate in VecTest may react with a nonspecific protein in the fecal, saliva, or tissue samples of the dead crows to create a false positive.

This preliminary investigation establishes the basis for more comprehensive research on the full capabilities of this test for WNV detection in birds, including the sensitivity of the test, the postmortem period for which the test is viable, and the effectiveness across a range of species. We suggest that VecTest may be a cost-effective, rapid field technique for WNV detection in dead American Crows in the early transmission season or in areas with low transmission rates, although confirmation of positives is suggested at this time. This assay may be a useful tool for epidemiologic studies of WNV transmission cycles involving American Crows and will help to provide an epidemiologic basis for vector control efforts, although further study is warranted.
